# UK Research Priorities for Electronic Cigarettes: A James Lind Alliance Priority Setting Partnership

**DOI:** 10.3390/ijerph17228500

**Published:** 2020-11-17

**Authors:** Abby Hunter, Louise Ross, Toto Gronlund, Sue Cooper

**Affiliations:** 1 Division of Epidemiology and Public Health, School of Medicine, University of Nottingham, Nottingham NG5 1PB, UK; 2 National Centre for Smoking Cessation and Training, Dorchester DT1 1RD, UK; lou_ross@yahoo.com; 3 The James Lind Alliance, National Institute of Health Research Evaluation, Trials and Studies Coordinating Centre, University of Southampton, Southampton SO16 7NS, UK; toto.jla@microwells.uk; 4 Division of Primary Care, School of Medicine, University of Nottingham, Nottingham NG7 2RD, UK; sue.cooper@nottingham.ac.uk

**Keywords:** e-cigarettes, prioritisation, tobacco policy

## Abstract

This study aimed to bring together people who smoke or vape, people who do not smoke and healthcare professionals to identify and agree priorities for electronic cigarette research in the UK. We carried out a priority setting partnership, guided by the methodology developed by the James Lind Alliance involving five key stages: initiation, consultation, collation, prioritisation and dissemination. A total of 765 people submitted 1887 questions that they wanted answered by research. Questions were organised into themes, merged and rewritten as summary questions, with 52 unique questions going forward to the prioritisation survey. Participants then ranked their top 10 questions. Following this ranking exercise, the top 26 were identified by selecting the most frequently prioritised questions adjusting for representative stakeholder group. These were put forward for discussion in the final prioritisation workshop, whereby the top 10 electronic cigarette research questions were agreed. The list of priorities identified will be of interest to researchers and funders of electronic cigarette research and will hopefully direct future research and funding calls. These priorities provide insight into the questions that matter to people who are using or concerned about e-cigarettes, including frontline professionals.

## 1. Introduction

Smoking is the leading cause of preventable morbidity and premature death. In the UK, 15% of the population smoke [1]. Intervening to help all people who smoke quit has major potential to improve current and future health of all patients who use the National Health Service (NHS). Smoking cessation interventions are, therefore, extremely cost-effective. Available NHS treatment includes Nicotine Replacement Therapy, varenicline, and bupropion, as well as behavioural support. Electronic cigarettes (e-cigarettes) are another type of quitting aid, although not yet available on the NHS. They have increased in popularity, and are the most popular aid to smoking cessation, with 37% of people who smoke using them for quit attempts [2], and approximately 3.6 million UK adults currently using them [3]. However, the current evidence base is small and only just emerging. There is much unknown, including the long-term effects. As the popularity of e-cigarettes has increased, so has the controversy associated with using them, and sensationalist media headlines may deter their use or promote stories without scientific backing.

E-cigarette research is a relatively new topic; therefore, it is essential to identify and address research priorities that are relevant to people who smoke or vape, and healthcare and public health professionals (HCPHP). Typically, academic researchers decide the questions that are addressed and investigated, but their priorities may not match those of patients and carers. There is often a mismatch between the types of interventions patients and clinicians wish to see evaluated. There is a need to ensure research is informed and guided by the needs of patients to avoid waste in terms of inadequate focus and outcomes [4]. Given that resources for research are limited, it is important for research funders to understand the priorities so future research can be targeted accordingly. The James Lind Alliance (JLA), established in 2004 by the National Institute of Health Research (NIHR), brings together patients, carers and clinicians, in Priority Setting Partnerships (PSP), to agree the most important uncertainties for particular health conditions [5]. It is one of the most established and pragmatic processes for prioritising health research questions. The JLA provides an established step-by-step methodology for managing and completing a PSP, ensuring all processes are accountable and transparent. To date, there have been 125 PSPs on a wide range of topics including maternal health, hearing problems, mental health and child health. To give an example of how the PSP process has impacted the direction of future research, if we take the topic of hearing research, there has been a PSP on hyperacusis (2018); mild to moderate hearing loss (2015) and tinnitus (2012). Of the top 10 tinnitus priority questions, five have been addressed by funded research and the other five are currently being addressed.

The Cochrane Tobacco Addiction Group (TAG) conducted a priority-setting stakeholder engagement project to identify where further research is needed in the areas of tobacco control and smoking cessation [6]. E-cigarettes were identified as a priority area for further research. The TAG study was broad in scope, whereas this study is narrower and focuses specifically on delving deeper into identifying and prioritising uncertainties in e-cigarette research, thus adding value to the TAG study by focusing on one specific area. The objectives of this PSP were to: (1) enable people who smoke or vape, people who do not smoke, and HCPHP to identify uncertainties in e-cigarettes in the UK, (2) agree by consensus on a prioritised list, (3) publicise the results and processes and (4) promote the priorities to researchers and funding agencies.

## 2. Materials and Methods

The e-cigarette PSP took place between June-2018 and September-2019. The JLA method (outlined in the JLA guidebook [7]) is open and transparent, involving five stages: initiation, consultation, collation, prioritisation and dissemination. The method uses an established and tried-and-tested process ensuring methodological transparency which minimizes the risk of bias.

### 2.1. Ethical Considerations

The people who take part in the surveys and priority setting stages of the work are not research participants. Thus, there is no requirement for ethics approval. The University of Nottingham Research Ethics Committee deemed this work not to require ethical approval. Completion of the surveys implied consent for submitted information to be included. Principles of ethical research were followed such as confidentiality.

### 2.2. Initiation

A steering group was convened with ”patients” (representing people who smoke and/or vape) and HCPHP. A typical PSP uses the term ”patient”; however, the steering group felt it was more appropriate to refer to people who smoke and/or vape as consumers. The steering group comprised four HCPHPs (a midwife, a stop smoking specialist, a respiratory consultant, a public health representative (PHE)), five consumers, three academics, and was chaired by a JLA advisor who supported the group throughout the process to understand the steps required. Each member of the Steering Group completed a ‘Steering Group Interests and Privacy form’, to create a culture of transparency in the group and help the JLA Advisor manage potential bias. Each member of the steering group brought with them knowledge of the topic, an understanding of the patient and clinician populations and access to networks of patients and clinicians. The role of the steering group was to coordinate the PSP and was responsible for a number of tasks, including publishing the initiative, overseeing the checking and collating of uncertainties and taking the final priorities to research funders. The steering group met face-to-face on three occasions with another four teleconferences during key stages of the study.

### 2.3. Consultation

Uncertainties about e-cigarettes were collected via a survey which consisted of an explanation of the study, basic demographic questions (age, gender and first part of their postcode), self-reported smoking status (e.g., current smoker/vaper; ex-smoker/vaper, or never smoker), whether they were a HCPHP and an open-ended question: *”What questions about electronic cigarettes would you like to see answered by research?”* In order to keep the survey as succinct as possible to encourage completion, we did not include further questions such as educational background or participants’ health literacy or understanding of research. It was felt that this information would not be necessary as we did not ask them to consider how a question would be answered or how a study may be designed, only to consider what the priority questions would be. Participants could submit as many questions as they wanted. Participants could contact the lead investigator if they wanted to be involved in the subsequent prioritisation stage.

The survey was circulated via steering group members, partner organisations (PHE, Cancer Research UK (CRUK), Action on Smoking and Health, National Centre for Smoking Cessation and Training, Royal College of Physicians, New Nicotine Alliance), and was available on the UK Centre for Tobacco and Alcohol Studies (UKCTAS) website. It was promoted on Facebook and Twitter. Efforts were made to disseminate the survey to target groups—for example, paper versions of the survey were taken to a local homeless centre. In addition to submitted uncertainties, we searched reviews of e-cigarettes for highlighted research uncertainties.

### 2.4. Collation

Uncertainties gathered in the consultation stage were reviewed, and out-of-scope questions were removed. A list was created of indicative uncertainties (i.e., similar questions were grouped together). The data were categorised using themes identified in the raw data. This process was managed by the lead investigator (with input from four steering group members) to ensure consistency in dealing with interrelated questions. Those that were difficult to assign to a theme were discussed with the steering group. Uncertainties that could be answered by published systematic reviews were removed from the process; these were identified by searching the Cochrane Library, Prospero and NHS Centre for Reviews and Dissemination databases. Uncertainty was confirmed if there was no review, an out-of-date review (over three years old) or a recent review with an equivocal answer. The collation process was closely monitored by the JLA advisor to ensure transparency and minimization of bias.

### 2.5. Prioritisation

The long list of indicative uncertainties was reduced into a more manageable list by asking participants to rank the questions according to those most important to them. Interactive card sorting software (www.optimalworkshop.com) was used to create an online survey whereby the list of indicative questions was presented in a randomised sequence for each participant. Participants read all questions and selected (drag and drop) the 10 questions they thought were most important for research, and ranked them in order of preference. To facilitate the process of identifying their top 10 questions, an intermediate ”questions of interest” box was included as a holding pen for questions they wished to consider. Participants could swap and change questions as often as they liked before submitting their final top 10. Participants could contact the lead investigator to express an interest in taking part in the final workshop.

The survey was not restricted to those who had completed the first survey, but was sent to all people who had registered an interest in participating in the next stage following the initial survey, and also circulated via steering group members, partner organisations and social media, as for the first survey.

Following this ranking exercise, the top 26 were identified by selecting the most frequently prioritised questions adjusting for representative stakeholder group. These were put forward for discussion in the final prioritisation workshop. The experience of the JLA [5] is that 26 is a sufficient number of uncertainties to identify the top 10.

A final consensus workshop with representatives from each stakeholder group was held in September 2019 to agree the top 10 research priorities for e-cigarettes. A blend of whole group and subgroup discussions was facilitated by three independent JLA advisers, two of whom had not been involved in the previous stages. The final top 26 uncertainties were shared with participants before the workshop in order for them to prioritise the questions in rank order and be prepared for discussions on the day. At the workshop, ground rules were agreed about confidentiality and respecting alternative viewpoints. Expectations were managed by highlighting that compromise would be necessary to accommodate the variety of perspectives.

On the day, participants were divided into three groups with mixed representatives from HCPHPs and consumers and an independent chair from the JLA. Each group worked together initially discussing their own personal priorities, and subsequently worked together to rank all questions. Cards with the indicative questions printed on them were laid out for the group to view, loosely ordered to reflect the initial discussion. In the second round, the rankings from the three groups were combined, and re-presented to the groups; the participants of these groups were mixed up so the groups had a different composition to the first round. The new groups each worked to review the combined ranking and adjust the priorities through facilitated discussion. The rankings from each group were again combined, and finally, all three groups came together to discuss and adjust this combined ranking until there was consensus on the top 10 priorities for e-cigarettes.

## 3. Results

### 3.1. Consultation and Collation Stage

**Figure 1:**
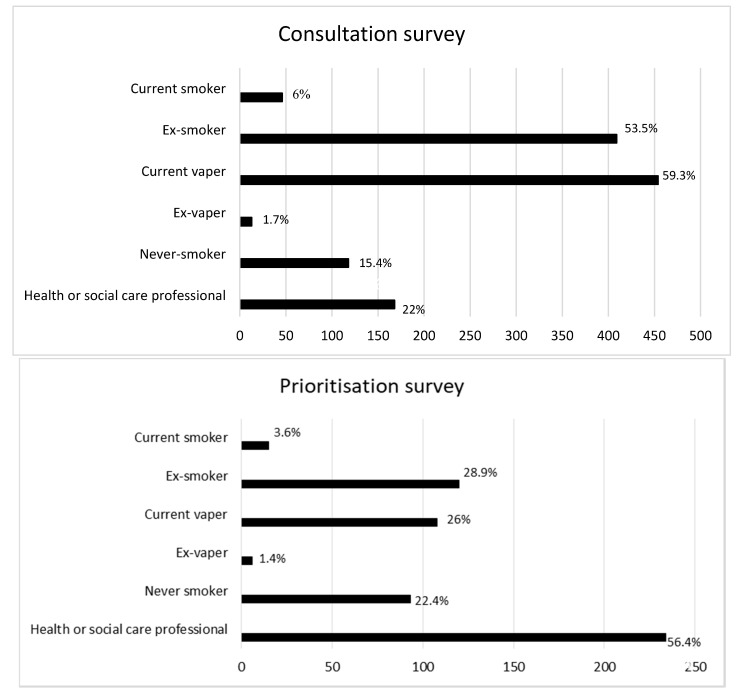
Smoking status of participants from the first round consultation survey and the second round prioritisation survey.

Overall, 765 participants (597 (78%) consumers/public, 168 (22%) HCPHPs) submitted 1887 uncertainties at the consultation stage. These could be anything they deemed important, including safety, effectiveness, different populations, supply or accessibility of e-cigarettes, environmental issues and related products such as snus and heated tobacco. Submitted questions per person ranged from one to 16. Participants ranged in age from 17 to 88 years old (mean age 47 years). Just over half of the participants were male (57.1%). Participants indicated their smoking status (see Figure 1) and could tick all that applied (e.g., current smoker/ex-smoker and current vaper).

HCPHP represented 168 participants. A fifth of these were stop smoking advisors. Other professions included nursing and midwifery, public health, medicine, dentistry, healthcare workers, mental health, pharmacy, physiotherapy, paramedics, occupational therapy, optics, audiology and social work.

Some submissions were just comments or statements with no underlying question, and these were excluded. After removal of out-of-scope submissions, nonquestions or statements, 1632 uncertainties remained. These were represented by 53 indicative uncertainties; however, the steering group agreed to remove one of these as it was not considered to be a true uncertainty based on the evidence *(“does vaping cause popcorn lung?”).* This left 52 indicative uncertainties that progressed to the ranking stage, representing 12 broad themes (health effects/safety, effectiveness, pregnancy, passive vape, regulation and policies, nicotine, prevalence, products, education and support, behaviours/accessibility, environmental and financial factors). The subthemes for each of these overarching themes can be seen in Supplementary Table S1. There were no differences in the types of questions submitted based on the smoking status of participants, other than the fact that some vapers asked specific questions about the devices—e.g., risk from burning cotton or coils; risks and benefits of salt nicotine vs. freebase nicotine; or other products such as heat-not-burn, snus or shisha.

The steering group agreed this was a suitably representative and manageable sized list to take forward to the next stage of the process. No other uncertainties were resolved by evidence of recent published systematic reviews.

### 3.2. Prioritisation

The ranking stage involved 415 participants (30.4% male) prioritising their top 10 from the list of 52 indicative uncertainties. Again, participants indicated their smoking status (see Figure 1). This time there was a greater proportion of respondents who were a HCPHP (56.4%). As smoking and/or vaping is common, if participants reported they were both a HCPHP and that they smoke or vape, they were classified as being a HCPHP for the summary statistics.

For the interim prioritisation, participants chose their top 10 questions from the list of 52 and ranked them in order of preference. These 52 questions and the frequency they were highly prioritised can be seen in Supplementary Table S2.

It was important to ensure the final indicative questions for the prioritisation workshop were representative of all groups—i.e., of equal significance to votes from across both stakeholder groups. The top 15 questions for HCPHPs, and the top 15 questions for consumers/public gave us a top 21 as nine of these questions were common in both groups. Each group had six questions that did not feature in the other group’s top 15. The steering group then voted for their top five questions from the next 10 most popular questions. This gave us a total of 26 questions for discussion in the final prioritisation workshop (see Table 1).

The final workshop to agree the top 10 research priorities (Table 2) had 23 participants from various regions of England and Scotland, including 15 HCPHPs (two of these were steering group members) and 8 non-HCPHPs (four of these were ”patient representatives” from the steering group). Two people observed the meeting, including one representative from CRUK, and one researcher.

During the workshop, each participant was given the opportunity to express their views on which questions they thought should be prioritised and to hear other people’s perspectives. Participants were able to debate and change their minds throughout the day. The research team observed that participants were keen to include as many topics as possible within the top 10 priorities—for example, ensuring there was a question related to pregnancy, and a question related to mental health within the top 10.

One change to the wording of a question was made following discussion and agreement from the workshop participants: the word ”prescribing” was changed to ”providing” in the question: *“Will prescribing e-cigarettes to pregnant smokers encourage smoking cessation, and reduce risk of relapse?”.* This word change was agreed with the lead investigator who felt this wording still represented the original respondents’ intent.

The top 10 priorities, (Table 2), were disseminated to all participants involved in the process via email. It was also shared on Twitter and the UKCTAS website. An infographic (Supplementary Figure S1) summarised the e-cigarette PSP process and the final top 10 priorities. Steering group members and partner organisations shared the final top 10 through their contact lists. The final top 10 were also presented to representatives from PHE and CRUK. The list of original submitted uncertainties, the 52 indicative questions and the final list of priorities taken to the workshop have been made available on and promoted through the JLA website [8].

## 4. Discussion

This PSP was conducted according to an independent and transparent process to identify and prioritise questions for research in which people who smoke or vape, people who do not smoke and HCPHPs were successfully brought together. To our knowledge, this is the first prioritisation exercise focusing specifically on e-cigarettes as a therapeutic aid.

The top ten research priorities include questions on long term safety; effectiveness for quitting smoking; policy; risks, benefits, barriers and facilitators to use in different populations including pregnancy and those with mental health issues; ingredients and testing; and the effects of second hand vape. This process has demonstrated numerous uncertainties surrounding e-cigarettes; such widespread uncertainty is likely to have an impact on the consistency of advice and information given by HCPHPs, and therefore patients’ confidence in this advice, as evidenced by the literature [9,10,11,12,13].

Most of the top 10 priorities of this PSP also align with those identified by the Cochrane Tobacco Addiction Group (TAG) [6]. Their priority setting tobacco control project identified e-cigarette research as one of the priority areas, particularly around the safety of the devices and effectiveness for smoking cessation (including cost-effectiveness). These priorities were identified in our top 10. However, TAG also prioritised research on effectively educating people about the risks and benefits of e-cigarettes, and this question was in our top 26.

CRUK [14] have also prioritised key areas for future e-cigarette research, of which all CRUK’s priority areas are covered within our top 10 questions, e.g., the drivers and external factors affecting use such as regulations and policies; outcomes including long-term effects, safety, effectiveness, relapse as well as in specific populations such as pregnant women and those with mental health conditions; psychological factors influencing how and why people use electronic cigarettes; and the biological effects of the ingredients, chemicals and flavourings in e-cigarettes.

It is reassuring, in this new field of research in e-cigarettes, consumers, HCPHPs and the research establishment are all on the same page when it comes to identifying priorities for future research. However, the 10th priority question in our PSP concerned the safety around second hand vape. This was not identified as a priority for TAG or CRUK, but it highlights the concerns the public and HCPHPs have and are perhaps seeking more assurance and evidence that second hand vape is harmless.

Our PSP has shown that there is a lack of research on e-cigarettes and many safety concerns among HCPHPs and consumers [15]. HCPHPs are giving inconsistent advice to patients [6], and there is a great deal of misleading information circulated on the topic.

There were no questions on supply or availability of e-cigarettes. Within the original uncertainties submitted, there were questions on how people access vaping equipment, including children and young people, and what the preferred sources are. However, these questions were ranked very low in the prioritisation survey, and therefore, were not discussed in the final workshop. However, the supply of e-cigarettes still remains an uncertainty, and preferred sources for accessing vaping equipment warrant further research.

With regard to other products such as heated tobacco, Swedish snus or shisha, there was one question asking what the risks and benefits of these products are and whether they should be treated the same as e-cigarettes (question asked by a vaper). However, this question was not ranked high enough in the prioritisation survey to be discussed in the workshop.

All submitted questions were verified against the evidence, but were still considered to be uncertainties, except one question on popcorn lung. The steering group agreed there was enough evidence that there is no risk of popcorn lung from vaping [16]. However, the fact this question was submitted 10 times, indicates this myth is widespread, and there are many myths around vaping that need addressing for the public [17].

For the other questions, there was often no systematic review evidence, or where there was a systematic review it lacked relevant studies to answer the question. For some questions, there were individual randomised controlled trials, and some participants in the final workshop argued that a question had already been answered because of available data from one trial. However, the JLA process requires a stronger evidence base, such as systematic review data, because a single trial can vary in quality and context, and a subsequent trial may report different results.

In order to keep the survey succinct to encourage responses, we did not ask many personal questions, so it is not possible to ascertain if we had responses from a wide range of ethnic populations, from areas of deprivation, or from specific populations—e.g., pregnant women, homeless population, prison population, those with mental health conditions or people suffering from other health conditions such as chronic obstructive pulmonary disease or lung cancer. However, efforts were made to ensure participants were as representative as possible to achieve a wide range of questions. The survey was distributed via the steering group’s broad network, to heads of maternity services, and to the general population via social media. Efforts were made to try to reach different populations—e.g., we succeeded in visiting a homeless shelter, but we faced difficulty reaching the prison population. Furthermore, we did not notice any difference in the types of questions submitted based on smoking status, besides the fact that some vapers asked questions specific to the devices, whereas non-smokers and smokers did not.

Additionally, this PSP was concerned with questions relevant to the UK population and organisations. However, many individuals from around the world submitted uncertainties, with 25 other countries represented. These questions were included if they were applicable to the UK. Nonetheless, it is likely that many of the questions will be applicable to an international audience—e.g., questions about safety and effectiveness including in different populations, long-term effects, effects on physical health and second hand vape. However, it is worth noting that there are wide variations in regulatory environments for e-cigarettes around the world. At least 14 countries have banned the sale and use of e-cigarettes, including Thailand, Brazil and Singapore. In the USA, at least five states have banned flavoured e-cigarettes (Massachusettes, New Jersey, New York, Rhode Island and California). Therefore, some questions may be dependent on national policy or regulatory environments and may vary by country (e.g., barriers and facilitators to use; the impact of restrictions on vaping or anti-smoking/vaping campaigns, and prevalence of use in different populations). Nonetheless, all questions in the top 10 will be relevant to an international audience but may need to be answered differently according to each country’s regulatory environment.

A recent publication has highlighted how JLA PSPs have transformed research, and the impact they have had [18]. One significant benefit that has arisen as a result of the many PSPs that have been completed is that the experience has changed relationships between funders and researchers. There is less emphasis placed on competition and greater emphasis on working together to achieve a common goal. There has been a shift in research funding towards the issues that matter most to patients, carers and healthcare professionals.

## 5. Conclusions

The list of priorities identified here will be of interest to researchers and funders of e-cigarette research and will hopefully direct future research and funding calls. These priorities provide insight into the questions that matter to people who are using or concerned about e-cigarettes, including front line professionals. Furthermore, many of these priorities also align with the issues identified by Cochrane TAG and CRUK.

## Figures and Tables

**Table 1:** The most frequently prioritised questions for both the healthcare professionals and non-healthcare professionals.

	Question	Both	HCP	Non-HCP
1	What are the long-term effects of vaping? Compared to smoking, ex-smokers, never-smokers and NRT use?	✓		
2	What are the short-term effects of vaping? Compared to smoking, ex-smokers, never-smokers and NRT use?	✓		
3	How do e-cigarettes compare to other treatments for stopping smoking, in terms of effectiveness, cost-effectiveness, long-term abstinence, and relapse to smoking?	✓		
4	How effective are e-cigarettes for smoking cessation in patients with mental health problems? And what effect do they have on mental health?		✓	
5	How safe are e-cigarettes in pregnancy, compared to smoking and NRT use?	✓		
6	What effect does vaping in pregnancy (and when breastfeeding) have on the health outcomes of the fetus and baby, compared to smoking? Including any long-term effects.	✓		
7	How effective are e-cigarettes in pregnancy for smoking cessation? And compared to other treatments?	✓		
8	Will prescribing e-cigarettes to pregnant smokers encourage smoking cessation, and reduce risk of relapse?		✓	
9	What effect does second hand vape have on adults (including pregnant women), children and animals, and how does this compare to second hand smoke?	✓		
10	What are the impacts of vaping on indoor and outdoor air quality, and how does this compare to other air pollutants?			✓
11	What impact do restrictions on vaping (e.g., including vaping in smoke free policies; age limits; tank size) have on smoking behaviour and smoking cessation? as well as perceptions of harm from vaping?			✓
12	What testing should be done on the flavourings, ingredients and devices to ensure they are safe?			✓
13	How addictive are e-cigarettes compared with regular cigarettes?			✓
14	What impact do flavourings have on e-cigarette usage, smoking behaviour and health, in adults and children, and smokers and non-smokers?			✓
15	What effect do the ingredients, chemicals and flavourings have on health, and how does this differ from cigarettes?	✓		
16	What are the views of healthcare professionals of e-cigarettes for smoking cessation (including in pregnancy)? And how can we improve knowledge?	✓		
17	How are e-cigarettes represented in the media? And what impact does this have on public perceptions, attitudes and behaviours?			✓
18	How can e-cigarettes be incorporated into a smoking cessation programme or treatment guidelines? How will this differ for different populations?		✓	
19	Can advice from healthcare professionals lead to better outcomes for smoking cessation if accurate information is provided about e-cigarettes?	✓		
20	How can hospitals better support patients to stop or reduce their smoking with the use of e-cigarettes?		✓	
21	What are the barriers and facilitators for e-cigarette use for smoking cessation? What different barriers may exist for those with mental health problems or heavily dependent smokers?		✓	
The Top 5 Voted for by the Steering Group from the Next 10 Most Frequent Responses				
22	What are the health effects of vaping for never-smokers and ex-smokers?			
23	What is the best way to educate adults and children about vaping, and to provide information about vaping products and device safety (including battery safety)?			
24	How can we motivate smokers to try e-cigarettes for smoking cessation?			
25	What effect does vaping have on mental health, including any risks and benefits? In adults, children and young people.			
26	What are the risks and benefits of nicotine consumption, and/or nicotine cessation?			

**Table 2:** Final prioritised and ranked uncertainties for electronic cigarette research.

Rank	Question
1	What are the long-term effects of vaping? Compared to smoking, ex-smokers, never-smokers and NRT use?
2	What effect do the ingredients, chemicals and flavourings have on health, and how does this differ from cigarettes?
3	What effect does vaping in pregnancy (and when breastfeeding) have on the health outcomes of the foetus and baby, compared to smoking? Including any long-term effects.
4	How effective are e-cigarettes for smoking cessation in patients with mental health problems? And what effect do they have on mental health?
5	What are the barriers and facilitators for e-cigarette use for smoking cessation? What different barriers may exist for those with mental health problems or heavily dependent smokers?
6	How do e-cigarettes compare to other treatments for stopping smoking, in terms of effectiveness, cost-effectiveness, long-term abstinence, and relapse to smoking?
7	What impact do restrictions on vaping (e.g., including vaping in smoke free policies; age limits; tank size) have on smoking behaviour and smoking cessation? as well as perceptions of harm from vaping?
8	What testing should be done on the flavourings, ingredients and devices to ensure they are safe?
9	Will prescribing/providing e-cigarettes to pregnant smokers encourage smoking cessation, and reduce risk of relapse?
10	What effect does second hand vape have on adults (including pregnant women), children and animals, and how does this compare to second hand smoke?

## Supplementary Materials

The following are available online at https://www.mdpi.com/1660-4601/17/22/8500/s1, Figure S1: Electronic Cigarette Priority Setting Partnership; Table S1: Number of submitted uncertainties for each theme and subtheme; Table S2: Rank of important questions based on frequency of the mode generated from the second round rating.
