# Supplementary material for: UK Research Priorities for Electronic Cigarettes: A James Lind Alliance Priority Setting Partnership
Source: Int J Environ Res Public Health. 2020 Nov 17;17(22):8500. doi: 10.3390/ijerph17228500 (PMC7697579; doi:10.3390/ijerph17228500)
Supplement: Supplementary file 1 [file ijerph-17-08500-s001.pdf]

International Journal of
Environmental Research and Public Health

MDPI

Supplementary Material

Table S1. Number of submitted uncertainties for each theme and sub-theme.

Theme	Total	HCP	Non HCP
**Health effects**	**611**	**161**	**450**
Long-term	212	57	155
Short-term	158	34	124
Physical effect	211	59	152
Mental health risks and benefits	10	4	6
Dual use	8	2	6
Effects on non-smokers	12	5	7
**Effectiveness**	**94**	**44**	**50**
Compared to other treatments	92	43	49
In patients with mental health issues	2	1	1
**Pregnancy**	**29**	**20**	**9**
Safety compared to other treatments	8	4	4
Effect on foetus	16	13	3
Effectiveness for quitting smoking	4	3	1
Prescribing e-cigs	1	0	1
**Passive vape**	**96**	**26**	**70**
Second hand vape effects	85	25	60
Impact on air quality	10	1	9
Stain furniture	1	0	1
**Regulation/ policies**	**70**	**14**	**56**
Restrictions on vaping	45	10	35
Vaping in prisons	2	2	0
Testing on flavourings	18	2	16
Effectiveness of anti-smoking/vaping campaigns	5	0	5
**Nicotine**	**118**	**28**	**90**
Addictiveness of e-cigs	51	14	37
Risks and benefits of nicotine consumption	31	4	27
How much nicotine is consumed when vaping	20	8	12
Nicotine strength	16	2	14
**Prevalence**	**39**	**26**	**13**
In adults, children, non-smokers and specific populations (e.g. pregnancy)	39	26	13
**Products**	**249**	**49**	**200**
Impact of flavours on usage	68	12	56
Risks and benefits of other products (HNB, Snus, Juul)	15	5	10
Effect of ingredients on health	56	13	43

Differences between devices	38	16	22
Comparison of salt nicotine and freebase nicotine	20	2	18
Risk from burning cotton coils	28	0	28
Temperature levels	24	1	23
**Education/ support**	**187**	**77**	**110**
Public perceptions of e-cigs	50	18	32
Views of healthcare professionals	28	11	17
How to educate adults and children about vaping	27	11	16
Representation of e-cigs in the media	16	3	13
Motivating smokers to try e-cigs	10	3	7
Incorporating e-cigs into treatment guidelines	14	11	3
Impact of financial incentives	5	2	3
Quitting vaping and avoiding relapse to smoking	19	8	11
Advice from healthcare professionals	10	6	4
Providing smoking cessation support in vape shops	5	2	3
Hospitals supporting e-cig use	3	2	1
**Behaviours/ accessibility**	**111**	**28**	**83**
Why do children and young people start vaping	10	3	7
Barriers and facilitators for e-cig use for smoking cessation	11	5	6
Why do non-smokers start vaping	7	1	6
Different ways of vaping and impact on health	28	4	24
Trajectories to and from vaping and smoking	7	3	4
Access to vaping equipment	8	1	7
Impact on experimentation with other drugs or addictive behaviours	40	11	29
**Environmental impact**	**14**	**0**	**14**
Production and disposal of e-cigs	14	0	14
**Financial**	**14**	**5**	**9**
Financial cost savings compared to smoking	10	4	6
Financial implications for the government	4	1	3
	**1632**	**478**	**1154**

Table S2. Rank of important questions based on frequency of the mode generated from the second-round rating. This table lists the 52 questions which were presented to participants (in a random order each time) and indicates the frequency that these were highly prioritised.

	Question	Frequency	Average Position
**1**	What are the long-term effects of vaping? Compared to smoking, ex-smokers, never-smokers and NRT use?	206	3.8
**2**	What effect does vaping have on physical health, including risks and benefits? e.g diabetes, cancer, oral health, respiratory conditions, allergies, digestion, physical activity, sexual health, eye health, weight, epilepsy and dementia.	199	3.9
**3**	What effect does vaping in pregnancy (and when breastfeeding) have on the health outcomes of the foetus and baby, compared to smoking? Including any long term effects.	169	4.1
**4**	How safe are e-cigarettes in pregnancy, compared to smoking and NRT use?	140	3.7
**5**	What effect does second hand vape have on adults (including pregnant women), children and animals, and how does this compare to second hand smoke?	131	5.4
**6**	How do e-cigarettes compare to other treatments for stopping smoking, in terms of effectiveness, cost-effectiveness, long-term abstinence, and relapse to smoking?	123	5.5
**7**	What effect do the ingredients, chemicals and flavourings have on health, and how does this differ from cigarettes?	121	5.1
**8**	What are the views of healthcare professionals of e-cigarettes for smoking cessation (including in pregnancy)? And how can we improve knowledge?	108	6.0
**9**	What testing should be done on the flavourings, ingredients and devices to ensure they are safe?	108	5.7
**10**	How addictive are e-cigarettes compared with regular cigarettes?	99	6.0
**11**	Will prescribing e-cigarettes to pregnant smokers encourage smoking cessation, and reduce risk of relapse?	98	4.7
**12**	How effective are e-cigarettes in pregnancy for smoking cessation? And compared to other treatments?	97	4.7
**13**	How can hospitals better support patients to stop or reduce their smoking with the use of e-cigarettes?	95	5.7
**14**	How can e-cigarettes be incorporated into a smoking cessation programme or treatment guidelines? How will this differ for different populations?	95	5.4
**15**	Can advice from healthcare professionals lead to better outcomes for smoking cessation if accurate information is provided about e-cigarettes?	89	6.0

**16**	What are the barriers and facilitators for e-cigarette use for smoking cessation? What different barriers may exist for those with mental health problems or heavily dependent smokers?	82	5.5
**17**	How effective are e-cigarettes for smoking cessation in patients with mental health problems? And what effect do they have on mental health?	77	5.5
**18**	What impact do restrictions on vaping (e.g. including vaping in smoke free policies; age limits; tank size) have on smoking behaviour and smoking cessation? as well as perceptions of harm from vaping?	73	6.1
**19**	What is the best way to educate adults and children about vaping, and to provide information about vaping products and device safety (including battery safety)?	70	6.2
**20**	How can we motivate smokers to try e-cigarettes for smoking cessation?	69	5.2
**21**	What are the health effects of vaping for never-smokers and ex-smokers?	67	5.3
**22**	What are the differences between all the available devices? Are any devices safer or more effective than others?	65	6.2
**23**	What are the impacts of vaping on indoor and outdoor air quality, and how does this compare to other air pollutants?	65	6.0
**24**	Why do never-smokers (including children) start vaping, and how/should we be discouraging never-smokers from starting vaping?	64	6.6
**25**	What are the short term effects of vaping? Compared to smoking, ex-smokers, never-smokers and NRT use?	63	4.7
**26**	What effect does vaping have on mental health, including any risks and benefits? In adults, children and young people.	62	5.0
**27**	How are e-cigarettes represented in the media? And what impact does this have on public perceptions, attitudes and behaviours?	60	5.6
**28**	What impact do flavourings have on e-cigarette usage, smoking behaviour and health, in adults and children, and smokers and non-smokers?	59	6.6
**29**	What impact has vaping had on the prevalence of smoking, initiation of smoking or experimentation with other drugs or addictive behaviours?	57	5.9
**30**	What is the best way to help people reduce nicotine strength, quit vaping and avoid relapse to smoking?	54	6.4
**31**	How much nicotine is consumed when vaping compared to smoking?	53	6.0
**32**	Why do children and young people start vaping? And how are they accessing e-cigarettes?	51	5.9
**33**	What are the risks and benefits of other products (e.g. heat-not-burn, swedish snus, juul, shisha) on the user and bystanders? And should these be treated the same as e-cigarettes?	51	5.5
**34**	How does nicotine strength affect probability of quitting smoking, and/or quitting vaping? And what impact does nicotine strength have	50	6.4

	on health?		
**35**	What are the different ways that people vape, and can this have an impact on health? (e.g. mouth to lung vs direct; frequency, nicotine strength).	48	6.5
**36**	What are the public perceptions of e-cigarettes and vape shops (including the views of children and young people), and do these views differ between different populations? And how can we improve understanding?	48	6.1
**37**	What impact would financial incentives of e-cigarettes have on smoking cessation? E.g. reduced price or free for certain populations.	47	7.7
**38**	What is the environmental impact of e-cigarettes, in terms of the production and disposal of units and packaging?	47	7.0
**39**	What are the risks and benefits of nicotine consumption, and/or nicotine cessation?	45	5.0
**40**	What are the risks and benefits of dual use compared with smoking only?	44	6.3
**41**	How effective are public health anti-smoking and/or vaping campaigns? What is the economic impact? And what effect do these have on different populations?	44	6.2
**42**	Are vape shops a suitable environment to provide smoking cessation support?	43	6.9
**43**	What is the prevalence of vaping (including dual use) in adults (including pregnant women), children and never smokers?	41	6.0
**44**	What is the risk from burning cotton or coils? Are some types better than others? How do we eliminate the metal?	38	5.8
**45**	What are the financial implications for the government, and the pharmaceutical and tobacco industry, of the introduction of e-cigarettes, in terms of loss of revenue from tax?	37	5.0
**46**	How does salt nicotine compare to freebase nicotine in terms of risks and benefits to health?	34	7.0
**47**	How do the different temperatures and power levels affect health? Is there an optimum temperature?	31	5.7
**48**	What impact does allowing vaping in prisons have on smoking behaviour and smoking cessation rates?	28	7.5
**49**	What are the trajectories to and from vaping and smoking? Including reasons for relapse to smoking, and accidental quitting?	25	5.8
**50**	What are the financial cost savings of vaping compared to smoking for the individual?	23	6.0
**51**	How are people currently accessing vaping equipment? What are the preferred sources? And how can we increase availability of vape products?	22	7.4
**52**	Does second hand vape leave a stain or smell on clothes and furniture?	13	6.7
